# Comment on Hasan et al. Clinico-Pathological Features and Immunohistochemical Comparison of p16, p53, and Ki-67 Expression in Muscle-Invasive and Non-Muscle-Invasive Conventional Urothelial Bladder Carcinoma. *Clin. Pract.* 2023, *13*, 806–819

**DOI:** 10.3390/clinpract16010009

**Published:** 2025-12-31

**Authors:** Stefan Harsanyi, Zuzana Varchulova Novakova, Stanislav Ziaran, Lubos Danisovic, Katarina Bevizova

**Affiliations:** 1 Institute of Medical Biology, Genetics and Clinical Genetics, Faculty of Medicine, Comenius University in Bratislava, Sasinkova 4, 811 08 Bratislava, Slovakia; zuzana.varchulova@fmed.uniba.sk (Z.V.N.); lubos.danisovic@fmed.uniba.sk (L.D.); 2 Department of Urology, Faculty of Medicine, Comenius University in Bratislava, Ruzinovska 6, 826 06 Bratislava, Slovakia; stanislav.ziaran@fmed.uniba.sk; 3 Institute of Anatomy, Faculty of Medicine, Comenius University in Bratislava, Sasinkova 2, 813 72 Bratislava, Slovakia; katarina.bevizova@fmed.uniba.sk

We read with great interest the article Clinico-Pathological Features and Immunohistochemical Comparison of p16, p53, and Ki-67 Expression in Muscle-Invasive and Non-Muscle-Invasive Conventional Urothelial Bladder Carcinoma by Hasan et al., who examined the expression of p16, p53, and Ki-67 in urothelial bladder carcinoma (UBC) in Egyptian patients [1]. We consider this topic worthy of research, as immunohistochemical (IHC) markers still remain a cornerstone of pathological examination.

However, the lack of association between p53 expression and tumor grade or stage prompted us to evaluate these markers in our larger cohort. Our study included 802 patients with UBC. We assessed clinicopathological features (grading, stage, multiplicity, recurrence) together with IHC examination of p53 and Ki-67. We observed strong correlations between grading and Ki-67 (ρ = 0.66) and p53 (ρ = 0.53), as well as staging with both markers: Ki-67 (ρ = 0.60) and p53 (ρ = 0.43). p53 and Ki-67 expression levels were moderately correlated (ρ = 0.56). These findings highlight that both markers increase steadily with tumor aggressiveness.

Results of chi-square tests based on our cutoffs (p53 ≥ 10%, Ki-67 ≥ 18%) confirmed significant associations between both markers and grade and stage (all *p* < 0.001). However, as the authors stated, the significance of differentiating between non-muscle-invasive bladder cancer (NMIBC) and muscle-invasive bladder cancer (MIBC) is not sufficiently clear. Due to this fact, we propose evaluating further cohorts at more discriminatory cutoffs (40% for p53 and 30% for Ki-67), which, although they lose some sensitivity, gain much specificity to discriminate between NIMBC and MIBC, also between low and high grades [2,3]. Comparisons of the standard and adjusted cutoffs for p53 and Ki-67 are presented in Table 1 and Table 2, respectively.

Together, these analyses suggest that, contrary to Hasan et al., p53 positivity is significantly associated with higher-grade tumors and deeper invasion, especially when analyzed in a large cohort with adjusted cutoffs. Ki-67 consistently demonstrates strong prognostic associations, confirming its role as a robust marker. We believe that the discrepancy may reflect differences in sample size, geographic population, and the use of cutoff thresholds. Importantly, our data advocate for the combined assessment of p53 and Ki-67, which, together, rather than alone, also provides better prognostic data in different types of cancer [4,5].

In conclusion, our findings support Ki-67 as a strong predictor of grade and invasion in urothelial carcinoma, and additionally reinforce p53 as a clinically relevant marker. We recommend conducting further multicenter studies that integrate continuous and categorical analyses to refine biomarker cutoffs and establish standardized protocols.

## Figures and Tables

**Table 1:** p53 cutoff at 10% vs. 40%.

Group	p53 (Standard)		p53 (Adjusted)		*p*-Value
	<10%	≥10%	<40%	≥40%	
LG	134	243	320	57	<0.001
HG	64	361	158	267	
NMIBC	161	372	403	130	<0.001
MIBC	36	232	74	194	

**Table 2:** Ki-67 cutoff at 18% vs. 30%.

Group	Ki-67 (Standard)		p53 (Adjusted)		*p*-Value
	<18%	≥18%	<30%	≥30%	
LG	197	180	293	84	<0.001
HG	51	374	81	344	
NMIBC	235	298	344	189	<0.001
MIBC	13	255	30	238	

## References

[1] Hasan A., Mohammed Y., Basiony M., Hanbazazh M., Samman A., Abdelaleem M.F., Nasr M., Abozeid H., Mohamed H.I., Faisal M. (2023). Clinico-Pathological Features and Immunohistochemical Comparison of p16, p53, and Ki-67 Expression in Muscle-Invasive and Non-Muscle-Invasive Conventional Urothelial Bladder Carcinoma. Clin. Pract..

[2] Caliskan I., Lu R., Szu Lyn Ding C., Sadala de Souza F., Perry A., Tihan T. (2024). Searching for a Cutoff Point for P53 Immunohistochemistry as Evidence of TP53 Mutations. Free Neuropathol..

[3] Li W., Lu N., Chen C., Lu X. (2023). Identifying the Optimal Cutoff Point of Ki-67 in Breast Cancer: A Single-Center Experience. J. Int. Med. Res..

[4] Faur I.F., Dobrescu A., Clim I.A., Pasca P., Prodan-Barbulescu C., Tarta C., Neamtu A.-A., Brebu D., Neamtu C., Rosu M. (2024). The Predictive Role of Serum Lipid Levels, P53 and Ki-67, According to Molecular Subtypes in Breast Cancer: A Randomized Clinical Study. Int. J. Mol. Sci..

[5] Dinu A., Aşchie M., Deacu M., Chisoi A., Enciu M., Cojocaru O., Vlad S.E. (2025). Clinico-Morphological Correlations with Ki-67 and P53 Immunohistochemical Expression in High-Grade Gastrointestinal Neuroendocrine Neoplasms. Gastrointest. Disord..

